# ZAP-70 Restoration in Mice by *In Vivo* Thymic Electroporation

**DOI:** 10.1371/journal.pone.0002059

**Published:** 2008-04-30

**Authors:** Magali Irla, Murielle Saade, Adrien Kissenpfennig, Lionel Franz Poulin, Lee Leserman, Patrice N. Marche, Evelyne Jouvin-Marche, François Berger, Catherine Nguyen

**Affiliations:** 1 INSERM U928, Université de la Méditerranée, Parc Scientifique de Luminy, Marseille, France; 2 Infection and Immunity, Centre for Cancer Research & Cell Biology (CCRCB), Queen's University Belfast, Belfast, United Kingdom; 3 Centre d'Immunologie de Marseille-Luminy, Université de la Méditerranée, INSERM U631, and CNRS UMR6102, Marseille, France; 4 INSERM, U823, Grenoble, France; 5 Université Joseph Fourier-Grenoble I, Institut Albert Bonniot, UMR-S823, Grenoble, France; 6 INSERM U836, Grenoble, France; 7 Université Joseph Fourier-Grenoble I, Grenoble Institut des Neurosciences, UMR-S836, Grenoble, France; Oregon Health & Science University, United States of America

## Abstract

Viral and non-viral vectors have been developed for gene therapy, but their use is associated with unresolved problems of efficacy and safety. Efficient and safe methods of DNA delivery need to be found for medical application. Here we report a new monopolar system of non-viral electro-gene transfer into the thymus *in vivo* that consists of the local application of electrical pulses after the introduction of the DNA. We assessed the proof of concept of this approach by correcting ZAP-70 deficient severe combined immunodeficiency (SCID) in mice. The thymic electro-gene transfer of the pCMV-ZAP-70-IRES-EGFP vector in these mice resulted in rapid T cell differentiation in the thymus with mature lymphocytes detected by three weeks in secondary lymphoid organs. Moreover, this system resulted in the generation of long-term functional T lymphocytes. Peripheral reconstituted T cells displayed a diversified T cell receptor (TCR) repertoire, and were responsive to alloantigens *in vivo*. This process applied to the thymus could represent a simplified and effective alternative for gene therapy of T cell immunodeficiencies.

## Introduction

The thymus is the primary site of generation of functional T lymphocytes. After maturation, T cells are released into the bloodstream and lymph fluid and then migrate to secondary lymphoid organs, mainly the spleen and lymph nodes. T lymphocytes play a crucial role in adaptative immune responses by recognizing antigens derived from pathogens or tumor cells. Adaptative immune responses may be impaired in several ways, including the natural mutations characterized by blocks in T cell development that have been described in mice and humans. These disorders are known collectively as SCIDs, because the clinical consequence is a devastating predisposition to infections and cancers. At least 9 different forms of human SCIDs have now been recognized and can be grouped according to genes involved such as γc, ADA, Artemis, ZAP-70, JAK-3, IL7Rα, CD45, RAG-1 and RAG-2 [1]. Gene therapy appears to be an appealing treatment by inserting corrective transgenes into abnormal cells [2]. Recombinant viruses are effective vectors for transferring genes into haematopoietic precursors. However, the main limitations reside in the possible development of opportunist cancers and in the potential toxicity in peripheral lymphocytes of the gene expression system controlled by retroviral long terminal repeats (LTRs) [3].

Gene therapy thus requires more efficient alternative therapeutic methods, using non-viral vectors in conjunction with an effective and safe physical process. Applying an electric field to cells and tissues significantly increases DNA uptake and gene expression [4]. Electroporation is usually performed by locally injecting DNA to the site of interest followed by the application of an electrical field. *In vivo*, electroporation has been already performed on several tissues, notably muscle and liver [5]. However, to our knowledge the thymus has never been electrotransfected. Therefore, *in vivo* electroporation of thymus appears to be a promising method to circumvent severe immunodeficiencies. Classical processes are aggressive using bipolar and even multipolar electrodes, often involving incisions, and the success of gene transfer may be compromised by aggressive surgical procedures [4].

Here, we describe a new method of electroporation without any incision, using a monopolar device to target the thymus *in vivo*. We stably electro-gene transferred thymocytes during their maturation in the thymus. We found the corresponding mature transfected T lymphocytes correctly localized in T zones of lymph nodes and spleen one month following treatment. We evaluated the usefulness of this approach to treat thymus-linked SCIDs using a murine model of ZAP-70 deficiency. ZAP-70 is a protein tyrosine kinase crucially involved in T cell differentiation. Its absence results in a SCID phenotype with a block at the CD4^+^CD8^+^ double-positive early stage of differentiation [6]. ZAP-70 gene transfer by *in vivo* thymic electroporation in ZAP-70^-/-^ mice results in the reconstitution of CD4^+^ and CD8^+^ single-positive thymocytes and the long-term detection of a high number of peripheral T lymphocytes displaying a diversified TCR repertoire and normal functional properties.

## Results

### A simple procedure to electroporate the thymus *in vivo*

**Figure 1:**
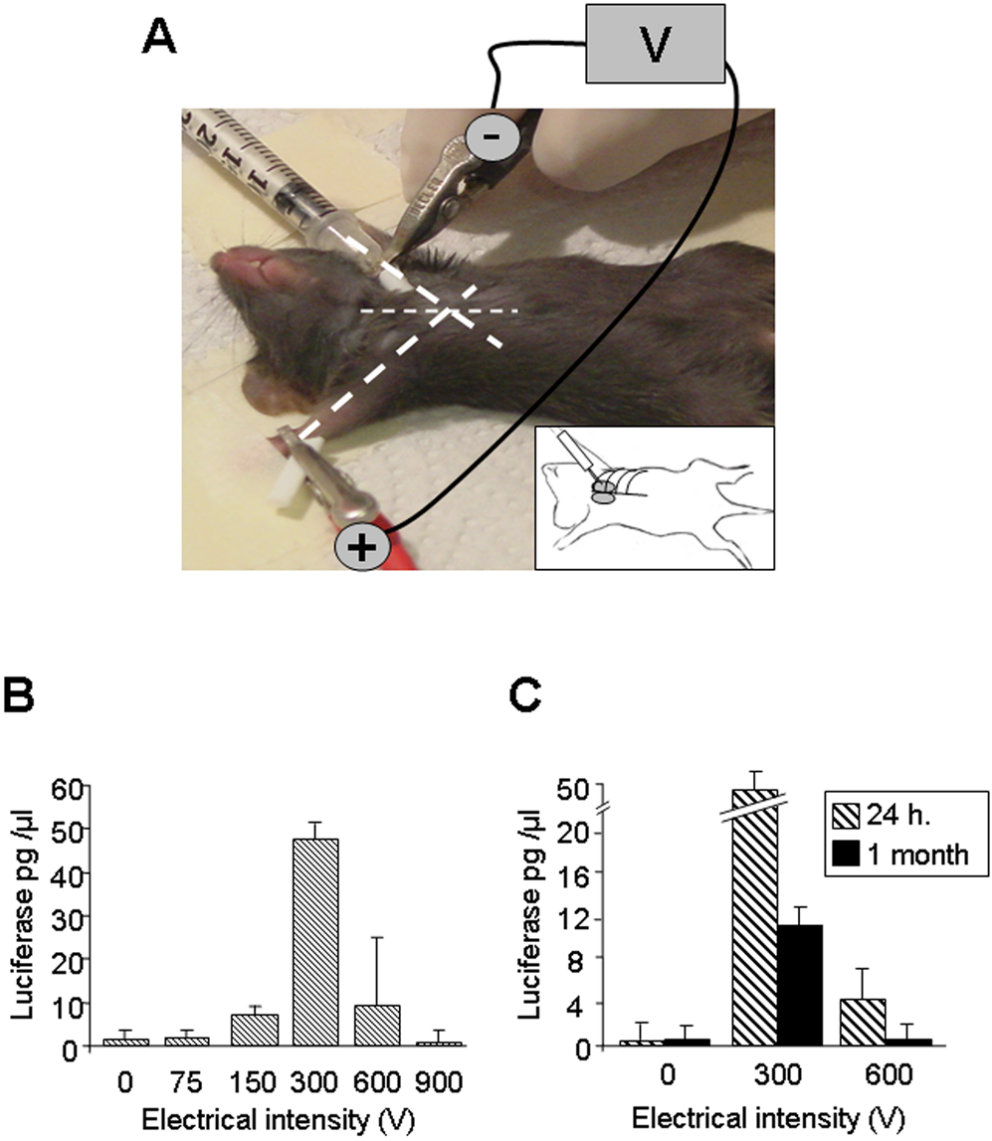
Monopolar electroporation of the thymus in anaesthetized mice. (A) Bold dotted lines indicate the angle of axis legs. The needle (optimal size: 3 mm) is inserted between the two first ribs, under the ribcage, following the angle of the paw parallel to the benchtop. The stimulating syringe, delivering electric pulses and plasmid DNA, represents the anodic electrode. The cathodic pole is represented by a crocodile clip attached to the paw of the side opposite to the injection point. The inset represents a schematic representation of the needle insertion through the ribcage to inject the thymus. (B) Luciferase activity in the thymus 24 hours after injection with or without electroporation. 10 µg of luciferase DNA plasmid in a volume of 10 µl was injected into each thymic lobe of 10 mice for 0 V, 10 mice for 300 V and 5 mice for all other voltages. Whatever the electric intensity applied, all mice survived. (C) Luciferase activity 24 hours (h) and one month after injection in the thymus without or with electroporation at 300 and 600 V, respectively. Four mice were analyzed for each condition.

The target organ is the mouse thymus. To transfect this deep organ directly *in vivo* without surgical intervention, we developed an electroporation procedure based on a monopolar device using a single needle as anode to inject the plasmid DNA and apply the electric field (Figure 1A). The needle size was calibrated according to the animal size and thymus localization. On an anaesthetized mouse, a crocodile clip corresponding to the cathode, establishes skin contact on the paw opposite to the injection site. We introduced the needle between the first and the second rib keeping the angle of the needle at 45° from the longitudinal axis (Figure 1A, inset). Thereafter, a volume of 10 µl of highly concentrated plasmid DNA was slowly injected in one thymic lobe and the current was immediately delivered. The second lobe was then treated in the same way. This technique allows electrotransfection of the thymus without affecting vital proximal organs such as the heart and lung, since all mice survived the intervention. Impact points on each thymic lobe were observed following electroporation (Figure S1A). Therefore, we established a safe simple procedure to electroporate the thymus *in vivo*.

### Optimization of electrical parameters

Previous in vivo electroporation analyses on the most widely targeted tissue which is skeletal muscle have shown that an effective conditions is 5 pulses of 1 Hz, at 100 µsec, with 10 µg of DNA [7]. Based on “muscle” parameters in combination with different voltages ranging from 0 to 900 V, we determine the optimal voltage to electrotransfect the thymus by transfecting the pCMV-luciferase (pCMV-luc) expression vector (Figure 1B). Maximal gene expression 24 hours after electroporation was achieved at 300 V. The amount of luciferase was increased 30-fold compared to the control condition (0 V). Moreover, we confirmed the transfection efficacy by *in vivo* bioluminescence (Figure S1B).

To assess whether these parameters result in a stable transfection, we investigated luciferase expression one month after injection or electroporation at 300 and 600 V of pCMV-luc plasmid (Figure 1C). We observed a complete absence of luciferase expression at 0 and 600 V. In contrast, at 300 V the luciferase expression was still detected although decreased five fold relative to 24 hours post-electroporation. This decrease can be partly explained by two physiological processes of the thymus. Firstly, the T cell maturation in the thymus takes about one month and mature T lymphocytes then migrate towards secondary lymphoid organs [8]. Secondly, approximately 90% of thymocytes are eliminated by apoptosis during selection mechanisms [9]. Altogether, these results showed that the *in vivo* thymic electroporation at 300 V leads to an efficient and stable transfection.

### Differentiating thymocytes are transfected *in vivo*

**Figure 2:**
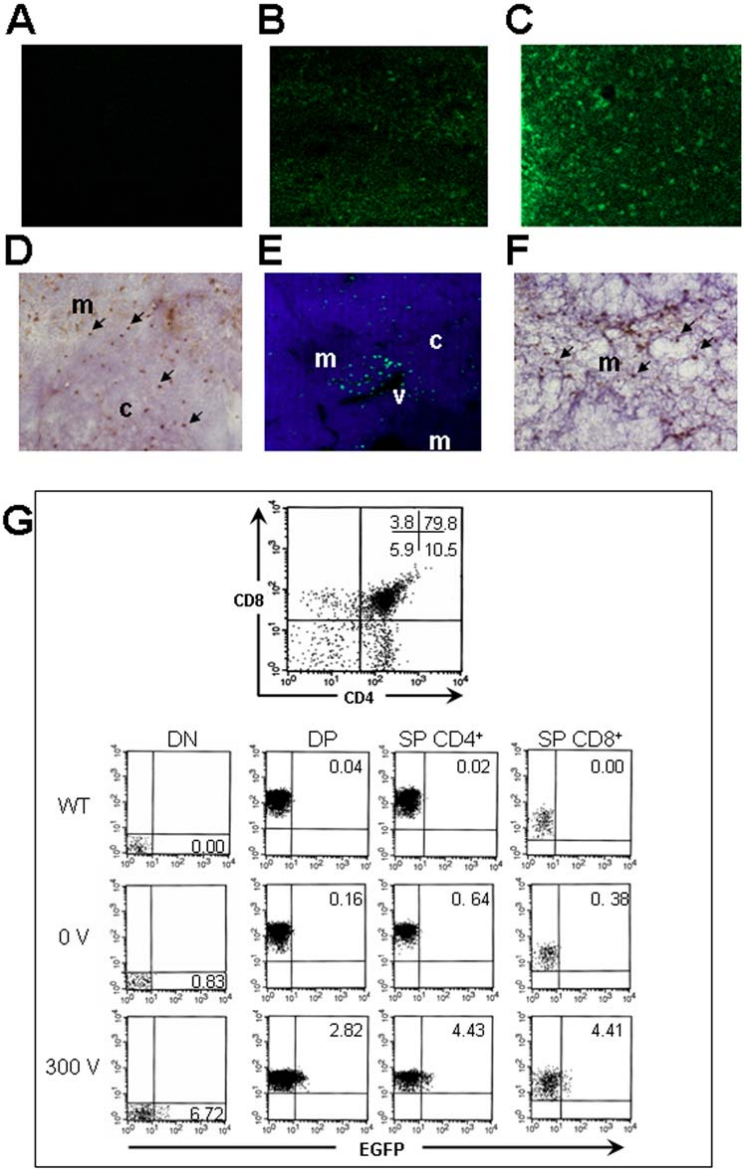
Distribution and immunophenotype of EGFP transfected thymocytes in electroporated thymi. Sections of thymi that were: (A) non injected; (B) injected without electroporation after 24 hours and (C) electroporated at 300 V after 24 hours were analyzed for the EGFP expression signal by confocal microscopy. Each image was captured and processed under the same conditions with a 10X objective. (D) 20X view of immunohistochemistry analysis using an anti-EGFP antibody of a thymus electroporated with the EGFP DNA plasmid at 300 V after 24 hours. (E) After one month, a nuclear counterstain (DAPI, blue) shows that these transfected cells are localized around blood vessels near the cortico-medullary junction (CMJ). (F) 20X view of immunohistochemistry analysis using an anti-EGFP antibody shows numerous transfected cells in the medulla after one month. Arrows indicate the EGFP expression signal. Abbreviations: c = cortex, m = medulla, v = venule. (G) Thymocyte profile stained with CD4 and CD8 monoclonal antibodies was analyzed by flow cytometry after 24 hours thymic electroporation. The percentage of cells within each quadrant is indicated. The different populations of thymocytes expressing EGFP protein in wild-type, 0 V and 300 V electroporated thymus were analyzed. The percentage of EGFP transfected cells is indicated within the quadrant of each population.

We determined the distribution of transfected thymic cells by confocal microscopy. 10 µg of pCMV-Enhanced Green Fluorescent Protein (EGFP) expression vector was injected or electroporated at 300 V (Figure 2). Detailed observation of thymic sections revealed no necrosis or any sign of tissue damage near the injection site, suggesting that the procedure was well tolerated (data not shown). As expected, sections of the non-injected thymus exhibited no EGFP signal (Figure 2A). In contrast, after 24 hours, a weak uniform staining was observed at 0 V (Figure 2B); while at 300 V the signal was stronger and numerous clusters of transfected cells were visualized (Figure 2C). These results were also confirmed by immunohistochemistry experiments (Figure 2D). One month later, the level of transfected cells strongly decreased, which is consistent with luciferase results described above (Figure 2E, F). Moreover, transfected cells were localized in several distinct clusters localized around venules of the cortico-medullary region suggesting that transfected cells could be thymocytes at the end stage of maturation on their way to be exported to the periphery.

Three independent experiments were performed to confirm this hypothesis, by co-staining lymphocytes using the CD3 marker which labeled nearly the whole thymocyte populations (DN to SP). We observed that 3.6±0.8% of T cells are transfected in the cortex and 1.9±0.7% in the medulla 24 hours after electroporation (Figure S2). After one month, we still detected 1.1±0.4% of T cells in the medulla and at the cortico-medullary junction, but none in the cortex. This observation is consistent with the fact that mature thymocytes congregate near blood vessels to be exported to the periphery [10].

To immunophenotype EGFP transfected thymocytes, we analyzed EGFP expression within the four main thymocyte subpopulations (CD4^−^CD8^−^ double-negative (DN), CD4^+^CD8^+^ double-positive (DP) stage, CD4^+^CD8^−^ and CD4^−^CD8^+^ single-positive (SP) by flow cytometry after 24 hours (Figure 2G). We found similar percentages of transfection obtained by confocal microscopy (6.72% of DN, 2.82% of DP and 4.4% of CD4^+^ and CD8^+^ SP cells). As expected, we did not observe any significant transfection in the 0 V condition. Moreover, we deduced that thymocytes in cell cycle may be preferentially transfected such as DN thymocytes that undergo extensive proliferation [11] and SP thymocytes that expand the newly selected T repertoire [12]. Finally, while thymocytes are difficult to transfect using a non viral system, our method allows their transfection with reasonable success, and the expression of the transfected gene is observed in the main thymocyte subpopulations.

### Transfected T cells are detected in secondary lymphoid organs one month after *in vivo* thymic electroporation

**Figure 3:**
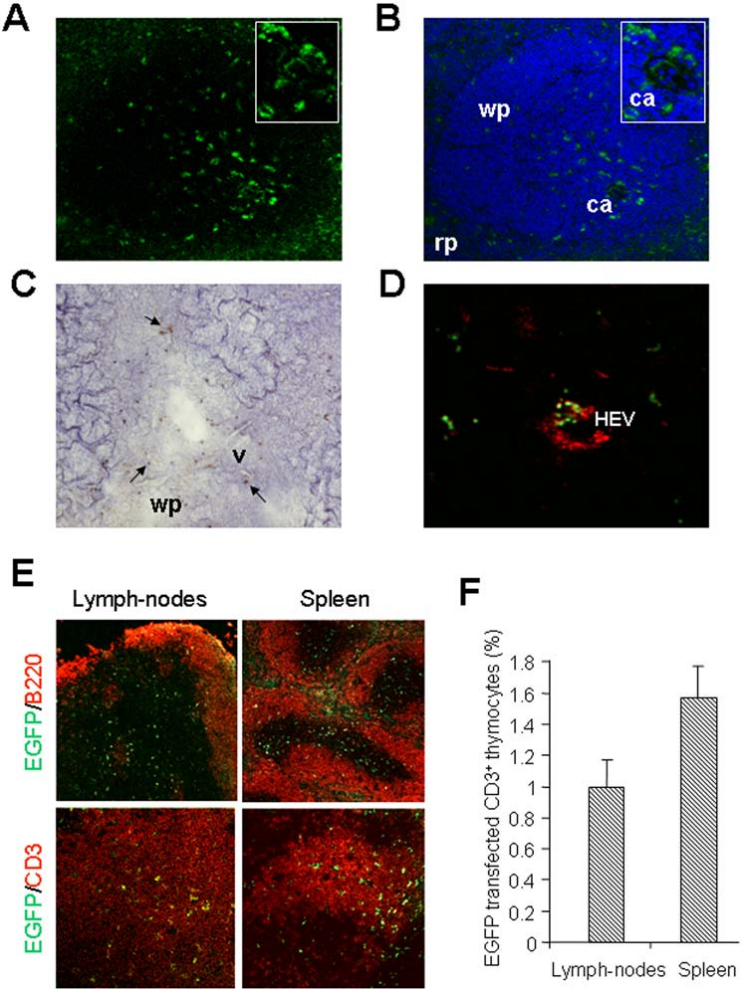
Detection of EGFP positive T lymphocytes in T zones of the spleen and lymph-nodes one month after thymic electroporation. (A) Fluorescence analysis at 100X view of a splenic internal region reveals EGFP positive cells. (B) A nuclear counterstain (DAPI, blue) shows that these positive cells are localized in the white pulp. Insets represent a higher magnification at 400X view of the central arteriole area (ca) where a cluster of EGFP positive cells is observed. (C) 100X view of spleen immunohistochemistry analysis using an anti-EGFP antibody shows the distribution of transfected cells (arrows). (D) EGFP positive cells are localized near high endothelial venules (HEVs) stained with an anti-PNAd antibody (red) in lymph-nodes. (E) Sections of lymph-nodes and the spleen were analyzed by confocal microscopy after staining with a B cell specific antibody (B220) and an anti-CD3 antibody that specifically reacts with T lymphocytes. Multiple representative clusters of EGFP transfected cells were found mainly in T zones of different lymph-nodes and spleen sections. (F) Percentages of EGFP transfected CD3 positive T lymphocytes present within T zones of lymph-nodes and the spleen were calculated as described in methods. The mean percentage and the standard deviation of 3 individual organs (n = 3) were calculated. Abbreviations: wp = white pulp, rp = red pulp, ca = central arteriole, v = vessel.

To determine whether transfected thymocytes develop into mature T cells, we analyzed spleen sections by two methods one month after thymic electroporation (Figure 3). Numerous EGFP cells were detected in the white pulp as shown by a DAPI counterstaining in confocal microscopy (Figure 3A, B), and confirmed by immunohistochemistry using an anti-EGFP antibody (Figure 3C). This observation is consistent with successful transfection of thymocytes in the thymus, and shows that transfected mature thymocytes were exported to the spleen. Moreover, a detailed observation showed that some transfected T cells are localized around a central arteriole suggesting that these cells are fresh immigrants [13] (Figure 3B; inset). The same analysis was also performed in lymph-nodes by labeling high endothelial venules with the PNAd antibody that recognizes the peripheral node addressin [14] (Figure 3D). We observed that incoming EGFP transfected T cells were found around venules.

These secondary lymphoid organs are organized in both T and B cell zones. To ensure that transfected T cells had correctly migrated into T cell zones, a co-staining analysis was performed on spleen and lymph-nodes sections by using anti-B220 and anti-CD3 antibodies to detect B and T cell areas, respectively (Figure 3E). B cell areas were mostly EGFP negative while T cell zones show numerous EGFP positive cells. We quantified the number of EGFP transfected lymphocytes using the CD3 marker and observed 1.0±0.2% of cells in lymph nodes and 1.5±0.2% in the spleen (Figure 3F).

Consequently, T cells were stably electroporated in the thymus, since significant numbers of transfected T lymphocytes were detected around homing sites and in T zones of secondary lymphoid organs one month later.

### Restoration of thymic development in ZAP-70^-/-^ mice after electroporation

**Figure 4:**
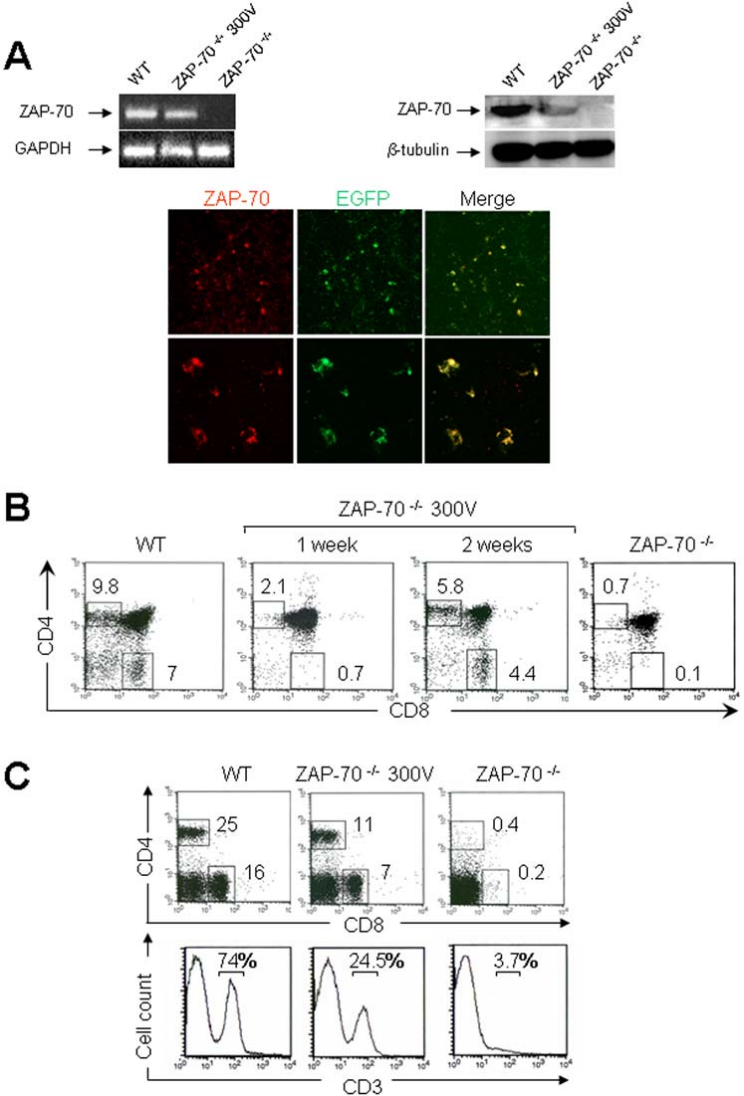
T cell reconstitution in ZAP-70^-/-^ mice following *in vivo* thymic electroporation with the pCMV-ZAP-70-IRES-EGFP DNA plasmid. (A) The upper panel shows ZAP-70 mRNA (left panel) and protein (right panel) levels in total thymocytes from WT, *in vivo* electroporated thymus at 300 V after 48 hours and in ZAP-70^-/-^ deficient mice assessed by RT-PCR and western-blot, respectively. The lower panel shows the distribution of ZAP-70 and EGFP proteins analyzed 48 hours after *in vivo* electroporation of the pCMV-ZAP-70-IRES-EGFP DNA plasmid by confocal microscopy (upper panel, 100X view). A higher magnification of transfected thymocytes is also presented (400X view). (B) Thymocyte profiles stained with CD4 and CD8 monoclonal antibodies were analyzed in WT and ZAP-70^-/-^ mice electroporated at 300 V with the pCMV-ZAP-70-IRES-EGFP DNA plasmid after one and two weeks as well as in ZAP-70^-/-^ mice. (C) three weeks after thymic electroporation, the presence of splenic T cells was monitored using CD4 and CD8 monoclonal antibodies (upper panel). Percentages of CD4^+^ and CD8^+^ T lymphocytes from WT and reconstituted mice as well as ZAP-70^-/-^ mice are indicated. The CD3^+^ T lymphocytes profile was also analyzed by using a CD3 monoclonal antibody (lower panel). The percentages of positively stained cells are indicated in each histogram.

We assessed the usefulness of this new approach to correct a thymus-linked immunodeficiency. We chose the murine model of Zeta-chain (TCR) Associated Protein kinase (ZAP-70) deficient SCID, which exhibits a relatively late block in T cell development at the CD4^+^CD8^+^ DP stage [6], [15]. We constructed the mouse ZAP-70/internal ribosome entry site/enhanced GFP (ZAP-70-IRES-EGFP) cassette under the transcriptional control of the cytomegalovirus (CMV) promoter. In this context, ZAP-70 and EGFP expression is concordant. Thymi of ZAP-70^-/-^ mice were electroporated with this expression vector at the optimal voltage of 300 V with a pulse length of 20 ms because longer pulse lengths improved transfection of muscle [16] as confirmed by our results for this gene in the thymus (data not shown). Transfection efficacy was evaluated after 48 hours by analyzing levels of ZAP-70 mRNA and protein in total thymocytes by RT-PCR and western-blot, respectively (Figure 4A, upper panel). As expected, the ZAP-70 mRNA (left panel) and protein (right panel) were clearly detected in wild-type (WT) thymocytes while no expression was observed in ZAP-70^-/-^ thymocytes. In contrast, total thymocytes from *in vivo* electroporated thymus expressed the ZAP-70 transgene, albeit more weakly than the endogenous protein in WT mice. ZAP-70 and EGFP ectopic expression were further studied by determining their distribution pattern by confocal microscopy (Figure 4A, lower panel). Numerous transfected cells that co-expressed ZAP-70 and EGFP proteins were detected in the thymus, confirming the successful transfection.

We then assessed the capacity of this transfection to restore thymocyte development. The CD4 and CD8 thymocyte profile was analyzed in WT and in electroporated ZAP-70^-/-^ mice after one and two weeks as well as in ZAP-70^-/-^ mice (Figure 4B). One week after electroporation, 2.1% of CD4 SP and 0.7% of CD8 SP were detected while after two weeks 5.8 and 4.4% were observed for CD4 and CD8 SP thymocytes, respectively. These results indicate that ZAP-70 expression driven from the electroporated pCMV-ZAP-70-IRES-EGFP DNA plasmid is capable of correcting the differentiation block in ZAP-70^-/-^ mice.

### Restoration of thymocyte development in ZAP-70-/- mice results in appearance of mature T cells in the periphery

In an attempt to determine whether *in vivo* gene transfer in ZAP-70^-/-^ thymi results in the appearance of mature T cells in the periphery, spleens were analyzed 3 weeks after thymic electroporation for the presence of CD4^+^ and CD8^+^ T lymphocytes (Figure 4C; upper panel). We detected considerable numbers of T lymphocytes in electroporated mice, reaching 11 and 7% for CD4 and CD8 T cells, respectively. Moreover, the same samples were also analyzed for the presence of CD3^+^ T lymphocytes (Figure 4C; lower panel). Similar results showing the appearance of a high level of T cells (24.5%) in electroporated mice compared to ZAP-70^-/-^ mice (3.7%) were obtained. Identical results were also obtained in lymph nodes (data not shown). We observed by confocal microscopy using an anti-CD3 antibody that a large numbers of transfected T lymphocytes were detected specifically in the splenic white pulp (Figure S3A, upper panel). These cells are ZAP-70 positive, as shown by western blot analysis (Figure S3A, lower panel). Moreover, we showed that newly generated T cells exhibit a characteristic CD44^hi^ CD62^lo^ activated phenotype occurring during T cell reconstitution of immunodeficient mice (Figure S3B) as found by Adjali and all [17]. Altogether, these data demonstrate that the correction in ZAP-70 expression by electroporation results in the presence of mature T cells correctly localized in the periphery.

### T lymphocytes exhibit a diversed receptor repertoire in reconstituted ZAP-70^-/-^ mice

**Figure 5:**
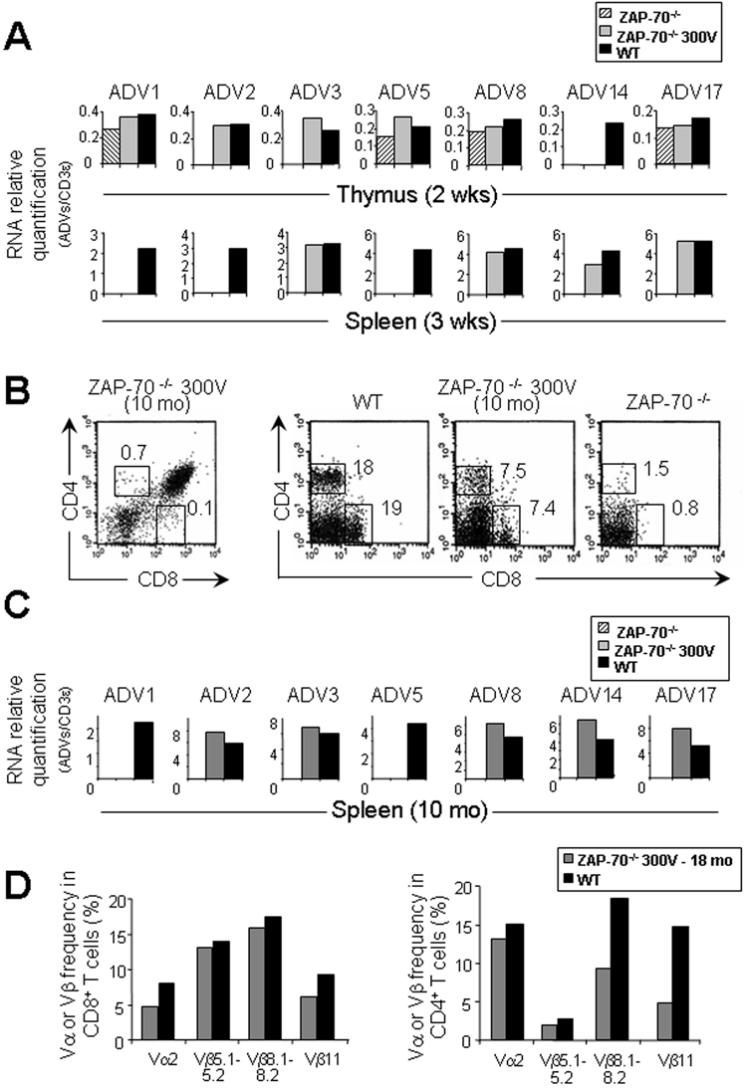
TCR repertoire diversity in ZAP-70^-/-^ electroporated mice. (A) Quantification of TCR V alpha transcripts in thymi and spleens between WT, untreated ZAP-70^-/-^, and ZAP-70^-/-^ mice electroporated at 300 V. Two and three weeks after electroporation, thymic (upper panel) and splenic (lower panel) cDNAs were respectively amplified using oligonucleotides specific for CD3ε and for a set of oligonucleotides specific for each of the V alpha genes in conjunction with a primer specific of the constant region of TCR alpha chain. Histograms correspond to a representative amplification of two separate experiments. (B) Thymocyte profile stained with CD4 and CD8 monoclonal antibodies was analyzed by flow cytometry after 10 months thymic electroporation (left panel). The persistence of CD4 and CD8 T lymphocytes in the spleen was monitored by flow cytometry 10 month after thymic electroporation (right panel). (C) Quantification of TCR V alpha transcripts in spleens between WT, untreated ZAP-70^-/-^, and ZAP-70^-/-^ mice with thymi electroporated at 300 V after 10 months. Splenic cDNA was amplified using oligonucleotides specific for CD3ε and for a set of oligonucleotides specific for each of the V alpha genes in conjunction with a primer specific of the constant region of TCR alpha chain. Histograms correspond to a representative amplification of two separate experiments. (D) TCRVα2 and indicated TCRVβ usage by splenic CD8 and CD4 T cells was evaluated by FACS analysis in eighteen month reconstituted mice compared to age-matched WT mice. Abbreviations: wks = weeks, mo = months.

To evaluate the quality of T cell repertoire in reconstituted mice, we used a quantitative PCR method that allows real time monitoring of PCR product appearance according to its relative abundance. We analyzed V alpha genes, which are highly expressed, such as V1, V2 and V3 and those that are less frequent, such as V5, V8, V14 and V17 [18]. The relative abundance of the transcripts was normalized to the CD3ε transcript. As expected in ZAP-70^-/-^ thymic samples, TCRV transcript products were not detectable or present in very low amounts (Figure 5A, upper panel) while in the spleen they were not detected (Figure 5A, lower panel). Interestingly, in reconstituted ZAP-70^-/-^ mice, all the TCRV transcripts tested were detected in thymus or spleen (Figure 5A). Moreover, we analyzed the size of the CDR3 of TCR transcripts for alpha chain in reconstituted mice, which showed polyclonal T cell population expressing a wide TCR repertoire as found in WT mice (Figure S4).

To monitor the persistence of T cells in reconstituted mice, we analyzed the CD4 and CD8 profile in the thymus and spleen ten months after thymic electroporation (Figure 5B). As expected, SP thymocytes were not detectable anymore in the thymus (CD4^+^: 0.7%; CD8^+^: 0.1%) (Figure 5B, left panel) while both types of T cells were still present in the spleen (CD4^+^: 7.5%; CD8^+^:7.4%) exhibiting a diversified TCR repertoire (Figure 5B, right panel, C). In addition, FACS analyses showed that TCR bearing Vα2 and several Vβ (Vβ5.1–5.2, Vβ8.1–8.2 and Vβ11) persist among mature CD8 and CD4 splenic T cells eighteen months following reconstitution (Figure 5D, S5). Representative FACS dot plots are also shown in Figure S5.

This indicates that T lymphocyte populations produced after *in vivo* thymic electroporation resulted from a wide variety of transfected thymocytes, which underwent thymic selection to generate a long-term diversified TCR repertoire.

### T lymphocytes exhibit normal functional properties in reconstituted ZAP-70^-/-^ mice

**Figure 6:**
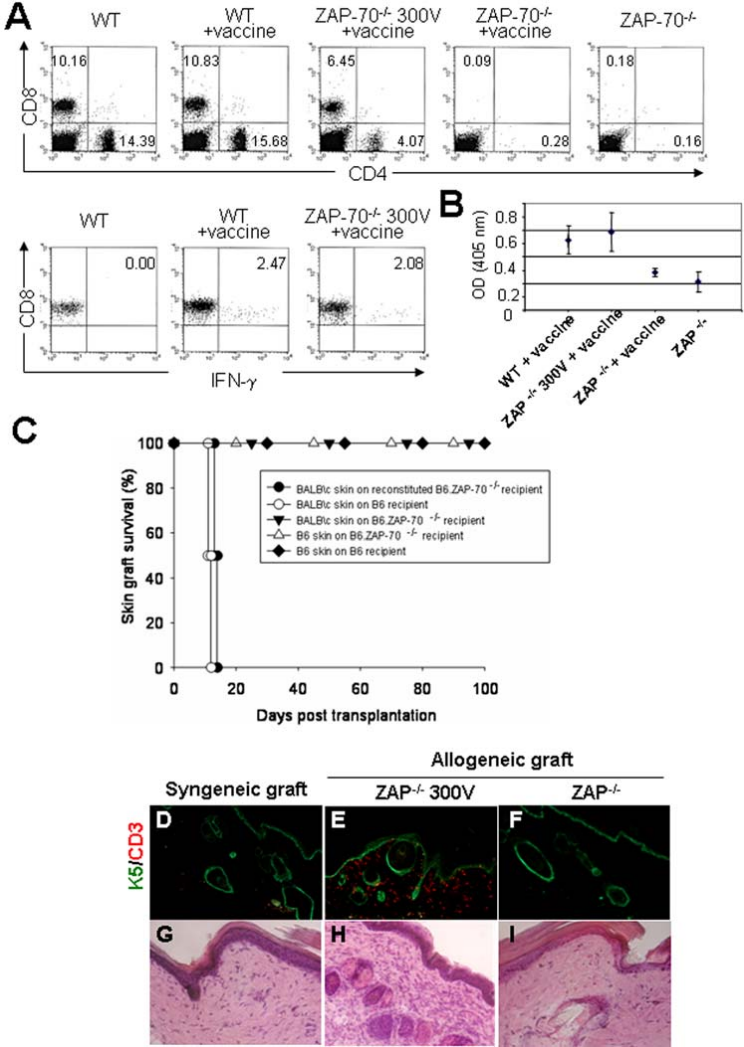
*In vivo* responsiveness of reconstituted T lymphocytes in reconstituted ZAP^-/-^ mice. One year old reconstituted mice were vaccinated with a plasmid encoding for the full length OVA. (A) The percentage of circulating T lymphocytes was evaluated in each indicated condition (upper panel) to monitor the long-term persistence of T cells in reconstituted mice. The function of CD8^+^ T cells was assessed by an antigen presentation assay of OT-I peptide and the percentage of produced IFN-γ was determined by intracellular FACS staining (lower panel). (B) The CD4^+^ T cell response following immunization was indirectly evaluated by measuring the level of anti-OVA IgG produced in reconstituted vs. WT mice (right panel). (C-I) Reconstituted C57BL/6 ZAP^-/-^ mice rapidly rejected fully MHC-mismatched BALB\c skin grafts. (C) Survival curves for allogeneic BALB/c skin grafts on C57BL/6 seven and sixteen months following reconstitution ZAP^-/-^ recipients (•) show rejection between day 10 and day 14, as fast as the allogeneic control (○). However, survival curves for syngeneic C57BL/6 skin grafts are similar in both the C57BL/6 WT (⧫) and the untreated ZAP^-/-^ recipients (▵) to allogeneic BALB/c skin grafts on untreated ZAP-70^-/-^ mice (▾), which don't show any graft rejection. Histological sections were performed of syngeneic (C57BL/6) skin that was grafted on WT C57BL/6 recipients (D, G) and allogeneic (BALB/c) skins which were grafted on a seven months electroporated ZAP^-/-^ recipient (E, H) and on an untreated ZAP^-/-^ recipient (F, I). These sections have been analyzed by hematoxylin-eosin (H/E, 200X view), to observed the presence of infiltrating cells (G, H, I), and by immunofluorescence (100X view), using antibodies against the epithelial marker Keratin 5 (K5, green) and the T lymphocyte marker CD3, to characterize these infiltrating cells (D, E, F). Contrary to the non-rejected skins (D, F, G, I) harvested 100 days after transplantation, the dermis of BALB/c allogeneic skin graft rejected at 50% by the reconstituted C57BL/6 ZAP70^-/-^ recipient contains numerous infiltrating cells (dark purple staining; H), characterized as CD3^+^ cells (E). TCR Vαn specific primer with a specific TCR constant alpha primer = ADVn

To evaluate *in vivo* the functionality of CD8 and CD4 T cells in long-term reconstituted mice, mice were vaccinated with DNA encoding the full-length ovalbumin (OVA) protein (Figure 6A, B). The percentage of circulating T lymphocytes was evaluated in each condition to monitor the persistence of T cells over sixteen months in reconstituted mice (Figure 6A, upper panel). Three weeks later, the anti-OVA CD8 response was analyzed on circulating T cells by *in vitro* stimulation with a specific OT-I peptide. In sixteen months reconstituted mice, CD8^+^ T cells exhibit a capacity similar to WT to produce IFN-γ, reflecting a normal CD8 response (Figure 6A, lower panel). CD4^+^ T cells functionality was evaluated by anti-OVA IgG titration (Figure 6B). Sixteen months reconstituted mice produced a similar antibody level as WT vaccinated mice, showing that CD4^+^ T cells have the capacity to give a cooperative help signal to B cells. All together, these results indicate that both CD8 and CD4 T cells possess normal functional properties.

To assess the capacity of the reconstituted C57/BL6 ZAP70^-/-^ mice to mount an allogeneic immune response, wild type and seven and sixteen month reconstituted mice were compared for their ability to reject fully histoincompatible BALB/c skin grafts (Figure 6C-I). The reconstituted mice rejected their allogeneic grafts between day 10 and day 14, as fast as the wild-type control (Figure 6C). Histological examination of rejected skin allografts revealed a marked difference between the seven month reconstituted C57/BL6 ZAP-70^-/-^ (Figure 6E, H) and untreated ZAP-70^-/-^ recipients (Figure 6F, I) as well as the syngeneic control (Figure 6D, G). Indeed, infiltration of CD3^+^ cells in the skin dermis were found in the former but were virtually absent in the latter. Thus, the reconstituted C57/BL6 ZAP70^-/-^ are able to develop an allogeneic response comparable to wild-type C57/BL6 mice. We can conclude that reconstituted C57BL/6 ZAP^-/-^ mice possess T lymphocytes with normal *in vivo* functional properties, enabling them to respond to an OVA DNA vaccine and rapidly reject fully MHC-mismatched BALB/c skin grafts.

## Discussion

Various methods based on the use of viruses as biological vectors have been developed over the years for the delivery of DNA. However, after decades of research and development, gene therapy still lacks a safe and efficient method permitting DNA delivery. The use of electric pulses to delive*r in vivo* therapeutic molecules to organs has seen rapid development over the last few years and represents an increasingly attractive non-viral method. The exponential increase in number of publications on the subject over the last 10 years testifies to this interest. By using bibliographic resources, using electroporation and gene therapy as key words, we found that electrotransfer has been applied to all easily accessible organs and also to internal organs when combined with surgery [19]. However, the thymus, the primary lymphoid organ in which several natural mutations have been characterized to induce a blockage in T cell development, has never been targeted by electro gene delivery for gene therapy.

The thymus is the organ in which haematopoietic precursors develop into competent T lymphocytes. To correct immunodeficiencies linked to natural mutations, transducing the therapeutic gene with a retroviral vector into hematopoietic stem cells (HSCs) has provided a proof of concept for gene therapy. However, because vectors are composed of retroviral portions including the LTR sequence that can activate additional viral sequences present in the vector, the development of alternative methods has been encouraged [2], [3], [20].

The method of electroporation we develop herein is based on a monopolar device to target the thymus *in vivo* without any incision. The classical procedure is simplified for two reasons: first, a single electrified needle is used to inject the plasmid and apply the electrical field, and second no surgical intervention is required. The method is also simpler and more economical than viral approaches. It allows the reduction of risks because only DNA is electrotransferred into the thymus. The electrical parameters that we defined lead to the targeting of thymocytes at various stages of differentiation. It has been shown that dividing cells are highly transfectable compared to quiescent ones, suggesting that DNA enters the nucleus upon the disassembly of the nuclear envelope during mitosis. Therefore, synchronizing the electrotransfer protocol with mitosis has been shown to improve gene delivery [21]–[23]. In our case, we do not need to synchronize since thymocytes are continuously in cell cycle. This biological process increases the efficiency of thymocyte electroporation within the thymus. Thus, these encouraging results led us to investigate the usefulness of this method for correcting T cell combined immunodeficiencies as proof of concept.

The efficacy of this approach is in large part due to the reconstitution of a SCID phenotype where thymocyte development is blocked at the CD4^+^CD8^+^ double-positive stage [6]. We demonstrated that a direct electroporation of the ZAP-70 gene into thymi of ZAP-70^-/-^ mice allows effective and rapid T cell reconstitution. Only two weeks later, CD4 and CD8 SP thymocyte differentiation occurred and significant numbers of T lymphocytes were present in the spleen and lymph nodes. Then, this non-viral approach achieved with nonsurgical intervention resulted in a significantly enhanced T cell reconstitution due to the direct targeting of the thymus. This observation is consistent with two recent studies which showed that intrathymic injection of either a ZAP-70 expressing lentiviral vector or WT HSCs enhances the kinetics of reconstitution [17], [24].

The efficacy of reconstitution by our method is also characterized in periphery by the detection of significant numbers of CD4 and CD8 T lymphocytes that exhibit a diverse TCR repertoire. The TCR repertoire observed in the reconstituted mice is characterized by a large pool of both alha and beta chains, although some discrete differences in the uses of V regions are observed in comparison to wild type mice. For instance in 10 months old reconstituted mice, Vα1 and Vα5 were not detected, and Vβ11 appeared under represented. The balance of the V uses in the repertoire is regulated during the ontogeny of the thymus [25], [26]. In as much as the transfection is carried out in young adult mice, thus later than the first steps of TCR alpha rearrangement in the thymus of wild type mice, the difference observed in the use of V genes in reconstituted mice may reflect changes in the regulation of TCR rearrangement and expression. Whatever the relative frequencies of the various V, the high degree of junctional variability at the CDR3 provides enough diversity to restore a fully functional TCR repertoire. Obviously in this model where thymocyte development is blocked at the DP stage, only transfected T lymphocytes can migrate and be functional at the periphery. Over the long-term, a peripheral partially diversified TCR repertoire was sufficient to provide functional T cells that mount *in vivo* immune responses to skin allografts and this response is specific to the alloantigens encountered (data not shown). Moreover, we monitored the persistence of long-lived T cells in periphery more than sixteen months after thymic electroporation and verified that both CD8 and CD4 T cells respond properly to an OVA DNA vaccine. Our data suggest that T cells persist for long periods in the periphery after their reconstitution by electro-gene transfer.

It is also important to note that the electroporation does not impair a normal immune response, as shown by this study and as well as by previous work on *in vivo* electroporation of splenic T lymphocytes [27]. However, the safety of gene transfer by electroporation in terms of potential insertional mutagenesis remains to be fully investigated [28]. We propose that thymic electroporation represents an interesting method for immunodeficiency gene therapy, which evidently requires optimization for clinical application, such as a systematic study to identify the optimal electropulsing sequence, injection periodicity and the *ad hoc* device for human electro-gene transfer.

We believed that this study is an exciting proof-of-principle that may advance the field of nonviral gene therapy. In addition, electrotransfer may also be used to efficiently deliver to the thymus a wide range of potentially therapeutic agents in addition to DNA, including proteins, oligonucleotides and siRNAs. An important feature of the monopolar electro-gene transfer resides in its potential application to other deep organs without surgery.

## Materials and Methods

### Mice

C57/BL6 WT (Iffa Credo), BALB/c WT (Charles River) and C57/BL6 ZAP-70^-/-^ (The Jackson Laboratory) [6] mice were bred and maintained under specific-pathogen-free conditions. All experiments were done in agreement with the French and European ethical rules. Thymic in vivo electro-gene transfer was performed at 3–6 weeks of age. Forty and twenty C57/Bl6 mice were transfected with pCMV-luc and pCMV-EGFP constructs, respectively. After 24 hours a subset of mice was sacrificed, thirty for the pCMV-luc and five for pCMV-EGFP for short term analysis, the other mice were sacrificed after one month for long-term analysis. T cell reconstitution experiments in ZAP-70^-/-^ mice were performed in five independent assays including at least five mice.

### Expression plasmids

Two expression vectors containing the cytomegalovirus (CMV) promoter inserted upstream of the coding sequence of the firefly luciferase (Promega), and enhanced green fluorescent protein (Clontech laboratories) were used for the optimization of electrical parameters. For T cell reconstitution experiments, the pCMV-ZAP-70 plasmid (kindly provided by AM Lellouch; CIML, Marseille, France) was used to insert the ZAP-70 gene into the pCMV-IRES-EGFP (Clontech laboratories). Each plasmid was amplified in DH5α bacteria andpurified on Qiagen columns by the lipopolysaccharide free method (Qiagen). A plasmid concentration of 5 µg/µl was maintained as a stock in 0.9% NaCl. The National Center for Biotechnology Information (NCBI) unigene cluster IDs (http://www.ncbi.nlm.nih.gov) for the ZAP-70 gene mentioned in the text is Mm.8038.

### Electroporation procedures

Animals were anaesthetized by intraperitoneal injection with a mixture of ketamine (100 mg/kg body weight; Imalgene 500; Rhone-Merieux) and xylazine (10 mg/kg body weight; Rompun 2%; Centravet). A 10 µl formulation containing 10 µg of pCMV-luc or pCMV-EGFP DNA was injected with an insulin syringe in each thymic lobe and the current was immediately delivered, with a standard square wave electroporator BTX T820 (BTX, Inc). 10 µg of DNA was tested with various values of voltage (0–900 V) and 5 pulses of 100 µsec. For T cell reconstitution experiments, a 10 µl formulation of 30 µg of pCMV-ZAP-70-IRES-EGFP DNA was injected in each thymic lobe and then an electrical current of 5 pulses of 20 ms at 300 V was immediately applied. At the end of the experiment the animals were kept warm until recovery.

### Thymic protein extraction and luciferase assay

The thymus was recovered and homogenized in 0.5 ml of lysis buffer (Promega) and centrifuged 10 min at 11000 rpm. Then, the supernatant was assayed for total proteins and luciferase activity with the luciferase assay kit from Promega for the measurement of light production during 10 sec interval in a TD20 luminometer (Turner design). The level of luciferase expression was quantified using commercial luciferase from *Photinus pyralis* (Boehringer-Mannhein).

### Bioluminescence imaging

BALB/c mice were anesthetized with 4% isofluorane before the intraperitoneal injection of 125 mg/kg body weight of luciferin (sodium salt; Promega). Ten minutes after the luciferin injection, photons emitted from luciferase within the animal and transmitted through its tissues were collected and integrated for a 5 min period images acquired using the NightOWL LB 981 CCD camera (Berthold Technologies GmbH & Co). Pictures were taken, onto which the pseudocolor image representing the spatial distribution of the detected photons was superimposed. The signal intensities from manually derived regions of interest (ROI) were obtained and data were expressed as photon flux (counts/s). Background photon flux was defined from a ROI of the same size placed in a non luminescent area nearby the animal and then subtracted from the measured luminescent signal intensity.

### Immunofluorescence

To identify the distribution and cells expressing the transfected gene, sections of 8 µm were prepared by cryosectioning after embedding the organs in OCT (Sakura Finetech) and mounted on glass slides. Sliced samples were kept in a humidified chamber and were not allowed to dry during staining. Sections were fixed with 4% paraformaldehyde (Sigma Aldrich) in phosphate buffer for one hour. Frozen sections were stained with anti-B220 hybridomas (RA6-6B2), anti-CD3 hybridomas (OKT3) (kindly provided by Dr. B Malissen, CIML, Marseille, France), unconjugated anti-peripheral LN addressin (PNAd) antibody (MECA-79) (Pharmingen) and anti-ZAP-70 (Santa Cruz biotechnology). Alexa 546–goat anti–rat IgG and Alexa 546–goat anti-rabbit (Molecular Probes) were used as secondary antibodies. As negative control, WT thymus, spleen and lymph-nodes were used in all experiments. Tissues were counterstained with 4′, 6′-diamidino-2-phenylindole (DAPI) at 1 µg/ml and mounted with Mowiol fluorescent mounting medium (Calbiochem). Skin sections were also stained with the anti-CD3 hybridomas (OKT3) and the anti-keratin 5 (AF138; Covance Research, Berkeley, CA).

### Microscopy and Digital Imaging

Fluorescent images were acquired by a Zeiss LSM 510 confocal microscope with a 515–525 nm bandpass filter set to view EGFP, a 560 nm longpass filter set to view alexa 546 and a 420 nm longpass filter set to view DAPI. All images were exposed using the same exposure time under the same magnification. Quantitative analysis of transfected cells distribution into thymus and secondary lymphoid organ sections was performed using ImageJ software (National Institutes of Health, Bethesda, Md) [29].

### Immunohistochemistry

Dessicated frozen sections were fixed with 4% paraformaldehyde (Sigma Aldrich) in phosphate buffer for one hour. Immunohistochemistry analysis was performed using the rabbit ABC staining system (Santa Cruz Biotechnology). Endogenous peroxidase activity was quenched by incubating slides for 10 min in 1% hydrogen peroxide diluted in deionized H_2_0. Sections were incubated for one hour in 1.5% blocking serum in PBS for blocking non-specific binding performed before antibody incubation. Rabbit anti GFP polyclonal antibody (BD Biosciences) incubation was performed for 30 min at room temperature. Single-color histochemical detection was performed using an avidin-peroxidase conjugate system, and the antibody-enzyme complex was visualized with 3′3′-diaminobenzidene (DAB). Incubation times were carefully monitored to prevent saturation, thus favoring visualization of differences in expression levels. Sections were counterstained using Mayer's hematoxylin solution (Sigma Diagnostics), dehydrated through graded ethanol and cover slipped using Eukitt mounting medium (Sigma Aldrich) for examination. Skin sections were also counterstained using hematoxylin-eosin solutions. Images were taken using Zeiss, axiophot 2 microscope with Nikon digital camera DXm1200.

### Flow cytometry

One to two million thymocytes were first incubated with the 2.4G2 hybridoma supernatant to block nonspecific binding of labeled antibodies. Then, the cells were stained with a mixture of PE-labeled CD4 and APC-labeled CD8 mAbs (BD Pharmingen). Viable cells were examined using a FACScalibur flow cytometer and data analyzed with Cell Quest software (Becton Dickson). For intracellular IFN-γ staining, blood cells stimulated overnight with OT-I OVA peptide (chicken OVA peptide 257–264 SIINFEKL) were first stained for PE-labeled CD4 and PerCP-labeled CD8 mAbs (BD Pharmingen) surface markers, followed by fixation in BD Cytofix/Cytoperm (BD biosciences). Staining was then performed with APC-conjugated anti-IFN-γ Ab (BD Biosciences) in 1X BD Perm/Wash buffer. Cells were washed in 1X BD Perm/Wash buffer twice before resuspension in FACS buffer and analysis. For the repertoire analysis, PE-labeled TCRVα2, TCRVβ5.1–5.2, 8.1–8.2 and 11 were used in combination with PerCP-labeled CD8 and APC-labeled CD4 antibodies (BD Pharmingen).

### RT-PCR

Total RNA was purified using TRIzol reagent (Gibco-BRL). Single-strand cDNA was synthesized by reverse transcription on 0.3–5 µg of total RNA using oligo(dT)25 and SuperScript II (Gibco-BRL) in a final volume of 20 µl. PCR reaction was performed in a PTC 200 Peltier Thermal Cycler (MJ research Inc) using 2 µl of the RT reaction product in a final volume of 50 µl, using the following conditions: 94°C for 3 min (1 cycle), 30 cycles of 94°C for 30 s, 66°C for 40 s, 72°C for 1 min, and 72°C for 10 min (1 cycle). Of the amplification product, 10 µl were resolved on a 1% agarose gel. The sequence primers used to detect specifically ZAP-70 are: forward primer: 5′-GCACATATGCACTGTCCCTGGTCTA-3′ and reverse primer 5′-GGGTCGCTGTAGGGACTCTCGTACA-3′ while EGFP was amplified with forward primer: 5′-AGTCCAAAGGCAGAGCCCCA-3′ reverse primer 5′-CGCTGTCACCTTCGAGGTTA-3′ and the housekeeping gene GAPDH with forward primer: 5′-AACGACCCCTTCATTGAC-3′ and reverse primer 5′-TCCACGAC ATACTCAGCAC-3′.

### Western-blots

To analyze the protein expression of ZAP-70, thymi and spleens were crushed and cytosolic proteins of T cells were isolated by using the Nuclear Protein Extraction Kit (Panomics). Protein concentrations were measured using the Pierce BCA protein assay. Samples were electrophoresed on 10% SDS-polyacrylamide gel and transferred to Nitrocellulose membranes (BioRad) following incubation with the rabbit polyclonal anti-ZAP-70 or the mouse polyclonal anti-β-tubulin (Santa Cruz biotechnology). Proteins were visualized using horseradish peroxidase-conjugated secondary antibody (Amersham Pharmacia Biotech,) and the enhanced chemiluminescence detection system (Pierce).

### Real time PCR

Total RNA from thymus or spleen samples was isolated using TRIzol reagent. Reverse transcription was done with SuperScript II RNase H^−^ kit (Life Technologies) according to the manufacturer's instructions. Each sample was tested for G3PDH as a housekeeping gene, for CD3ε to determine T lymphocyte levels and for several T cell receptor variable alpha genes (TCRV) to evaluate the diversity of T cells. PCR of cDNA sample was carried out on a Light Cycler apparatus (Roche Diagnostics) under the following conditions: 95°C 10 min, 30 cycles (95°C 15 sec, 59°C 10 sec, 72°C 15 sec). The amplification efficiencies of the different PCR reactions were determined as described^27^. The values for the yield ranged from 85 to 87% among the different primer combinations. Melting curves of PCR products were determined according to the manufacturer's instructions (Roche Diagnostics). The specificity of the unique amplification product was established by melting curve analysis and by migration on agarose gels. Each sample was analyzed in duplicate in separate experiments. For CDR3 length distribution, TCRValpha1 diversity analysis was done using a TCR VA1 specific primer together with a specific TCR constant alpha primer as described^27^. Run off extensions were performed with the following internal TCR constant alpha specific fluorescent anti-sense primer (5′CCATGGAATCTGGAACGTTCATC3′).

### Immunization and ELISA techniques

For DNA vaccination, mice were immunized in inguinal lymph node [30] with 10 µg of plasmid pDNK-OVA encoding the full-length OVA (kindly provided by Dr. P. Schuler). Plastic plates with 96 flat-bottomed wells were coated overnight at 4°C with 10 µg/ml of OVA to allow for the selective detection of high-affinity, OVA-specific IgG antibodies in the tested sera. Plates were washed with PBS, 0.05% Tween-20 and then blocked with 1% BSA+5% sucrose. After washing, dilutions of the test sera were added and incubated overnight at 4°C. Bound antibody was detected using anti-mouse gamma specific Alkaline phosphatase conjugated IgG (Amersham Pharmacia Biotech). Plates were developed with p-nitrophenyl-phosphate (Sigma-Aldrich), and absorbance was read at 405 nm.

### Skin grafting

Grafting tail skin onto the left flank of recipients was performed as previously reported [31], [32]. Briefly, skin grafts of approximately 1 cm diameter were prepared from tails of female mice and grafted onto the flanks of female recipients according to an adaptation of the method of Billingham and Medawar [33]. Petroleum gauze was placed over the graft, and sticking plaster was applied around the trunk. The bandages were removed after 10 days, and the grafts were monitored daily until day 30 and then every 2 days. Control syngeneic grafts (C57BL/6 donor skin on C57BL/6 recipient) always remained in excellent condition for more than 100 days.

## Supporting Information

Figure S1 Analysis of the thymus after monopolar electroporation. (A) The picture shows the healthy physical state of vital organs close to the thymus after injection and electroporation. Impact points in each thymic lobe are indicated by arrows. T: Thymus, H: Heart and L: Lung. The pCMV-luc plasmid was injected (0 V) or electroporated at 300 V and the luciferase expression was analyzed 24 hours later. In vivo bioluminescence indicated clear expression localized at the thymic site level. (B) Representative bioluminescence imaging of a mouse with an in vivo electroporated thymus with a luciferase DNA plasmid at 300 V in comparison to the 0 V condition. Levels of luminescence are indicated by false colors on a scale of 0 to 6000 units.
(1.47 MB TIF)

Figure S2 Efficacy of thymocyte transfection after in vivo thymic electroporation. (A) Characterization of transfected cells by co-staining analysis with an anti-CD3 antibody in the thymus 24 hours and one month after thymic electroporation. Insets correspond to a higher magnification of EGFP transfected CD3 positive thymocytes. (B) The histogram shows percentages of EGFP transfected CD3+ thymocytes present within the cortex and the medulla 24 hours after electroporation and in the medulla/CMJ one month later. The mean percentage and standard deviation of 3 individual thymi (n = 3) were calculated.
(2.32 MB TIF)

Figure S3 T lymphocytes exhibit an activated phenotype in reconstituted ZAP-70-/- mice. (A) The presence of EGFP T lymphocytes in reconstituted ZAP-70-/- mice was analyzed on splenic sections using a CD3 monoclonal antibody by confocal microscopy (upper panel). A nuclear counterstain (DAPI, blue) shows that transfected T lymphocytes are correctly localized in the white pulp (200X view). A higher magnification of transfected T lymphocytes was also presented (630X view). The lower panel shows a Western blot analysis of ZAP-70 protein levels in total thymocytes from spleens in WT, ZAP-70-/- deficient mice and in two weeks electroporated mice at 300 V (B) The phenotype of T lymphocytes was determined by using anti-CD62L and CD44 antibodies. The percentage of positively stained cells is indicated in each histogram.
(2.63 MB TIF)

Figure S4 The sizes of the CDR3 were analysed by PCR followed by gel electrophoresis. The figure displays representative experiments of the CDR3 analysis of TCR transcripts for alpha chain bearing Valpha1. The relative intensity of the bands (y axis) was plotted as a function of the migration time in the electrophoresis (x axis) which is proportional to the size of CDR3. In each profile, the peaks corresponding to the reading frame are indicated. In WT, the spectral distribution of the sizes is representative of polyclonal composition of the TCR Valpha1 transcripts. In ZAP-70 deficient mice, no TCR transcripts are found in the spleen, whereas a few peaks of TCR Valpha1 transcripts are detected in the thymus, which are issued from non productive transcription known to take place in these mice. After electroporation of ZAP-70 plasmid (ZAP-70-/- 300 V), TCR transcripts bearing 1αV are detected in the thymus as well as in the spleen. The spectral distributions of the CDR3 sizes are, and on the one hand, similar in the thymus and the spleen of ZAP-70-/- 300 V electroporated mice, and on the other similar for WT and ZAP-70-/- 300 V electroporated mice. Thus, ZAP-70-/- 300 V electroporated mice are reconstituted with a polyclonal population of T cells which expressed a wide repertoire of TCR.
(0.61 MB TIF)

Figure S5 Representative dot plots of TCRVα2 and indicated TCRVβ usage in splenic CD8 and CD4 T cells populations.
(1.57 MB TIF)
