# Supplementary figures and images for: ZAP-70 Restoration in Mice by In Vivo Thymic Electroporation
Source: PLoS One. 2008 Apr 30;3(4):e2059. doi: 10.1371/journal.pone.0002059 (PMC2323614; doi:10.1371/journal.pone.0002059)

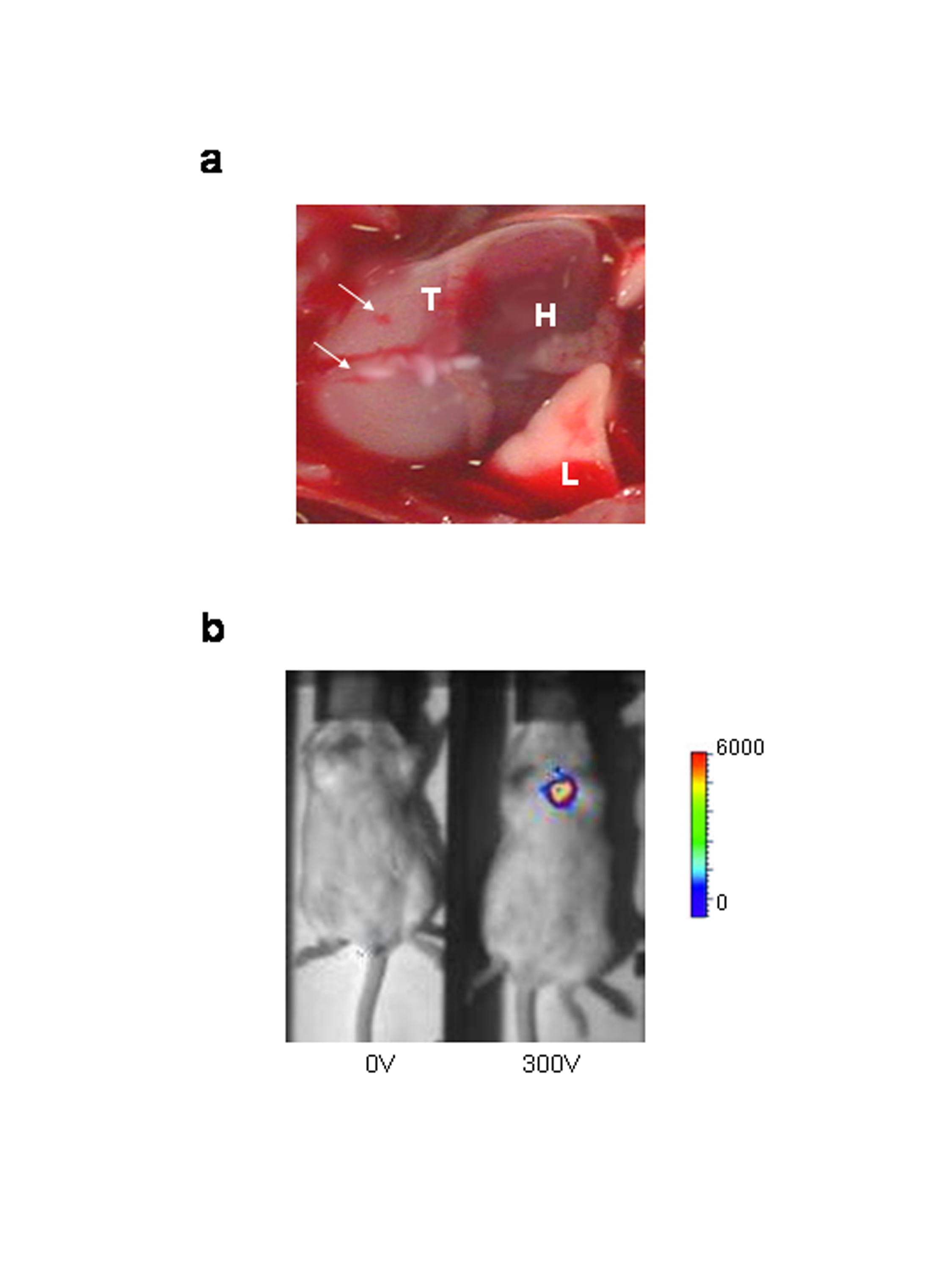

Supplement: Figure S1 — Analysis of the thymus after monopolar electroporation. (A) The picture shows the healthy physical state of vital organs close to the thymus after injection and electroporation. Impact points in each thymic lobe are indicated by arrows. T: Thymus, H: Heart and L: Lung. The pCMV-luc plasmid was injected (0 V) or electroporated at 300 V and the luciferase expression was analyzed 24 hours later. In vivo bioluminescence indicated clear expression localized at the thymic site level. (B) Representative bioluminescence imaging of a mouse with an in vivo electroporated thymus with a luciferase DNA plasmid at 300 V in comparison to the 0 V condition. Levels of luminescence are indicated by false colors on a scale of 0 to 6000 units. (1.47 MB TIF) [file pone.0002059.s001.tif]

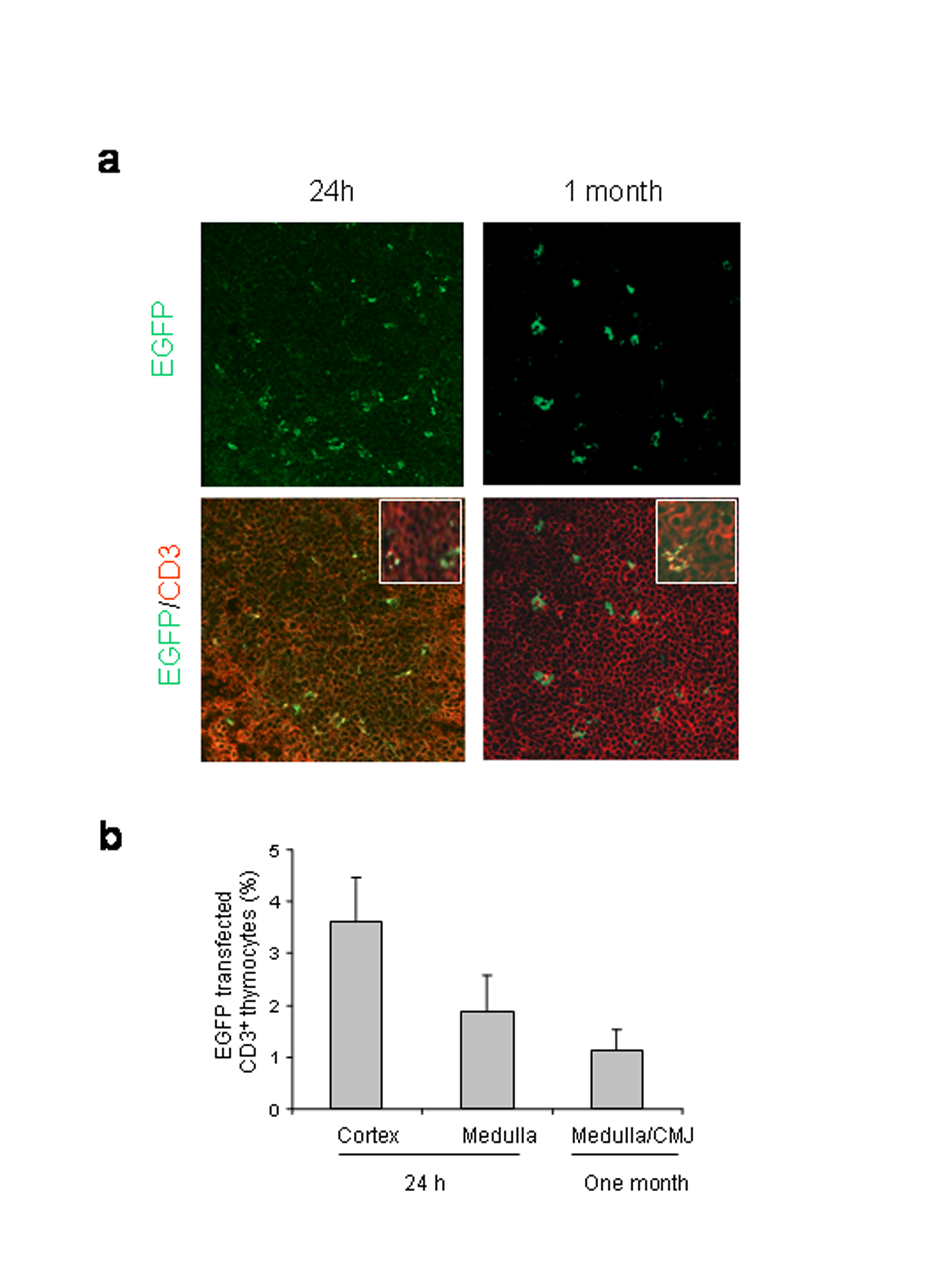

Supplement: Figure S2 — Efficacy of thymocyte transfection after in vivo thymic electroporation. (A) Characterization of transfected cells by co-staining analysis with an anti-CD3 antibody in the thymus 24 hours and one month after thymic electroporation. Insets correspond to a higher magnification of EGFP transfected CD3 positive thymocytes. (B) The histogram shows percentages of EGFP transfected CD3+ thymocytes present within the cortex and the medulla 24 hours after electroporation and in the medulla/CMJ one month later. The mean percentage and standard deviation of 3 individual thymi (n = 3) were calculated. (2.32 MB TIF) [file pone.0002059.s002.tif]

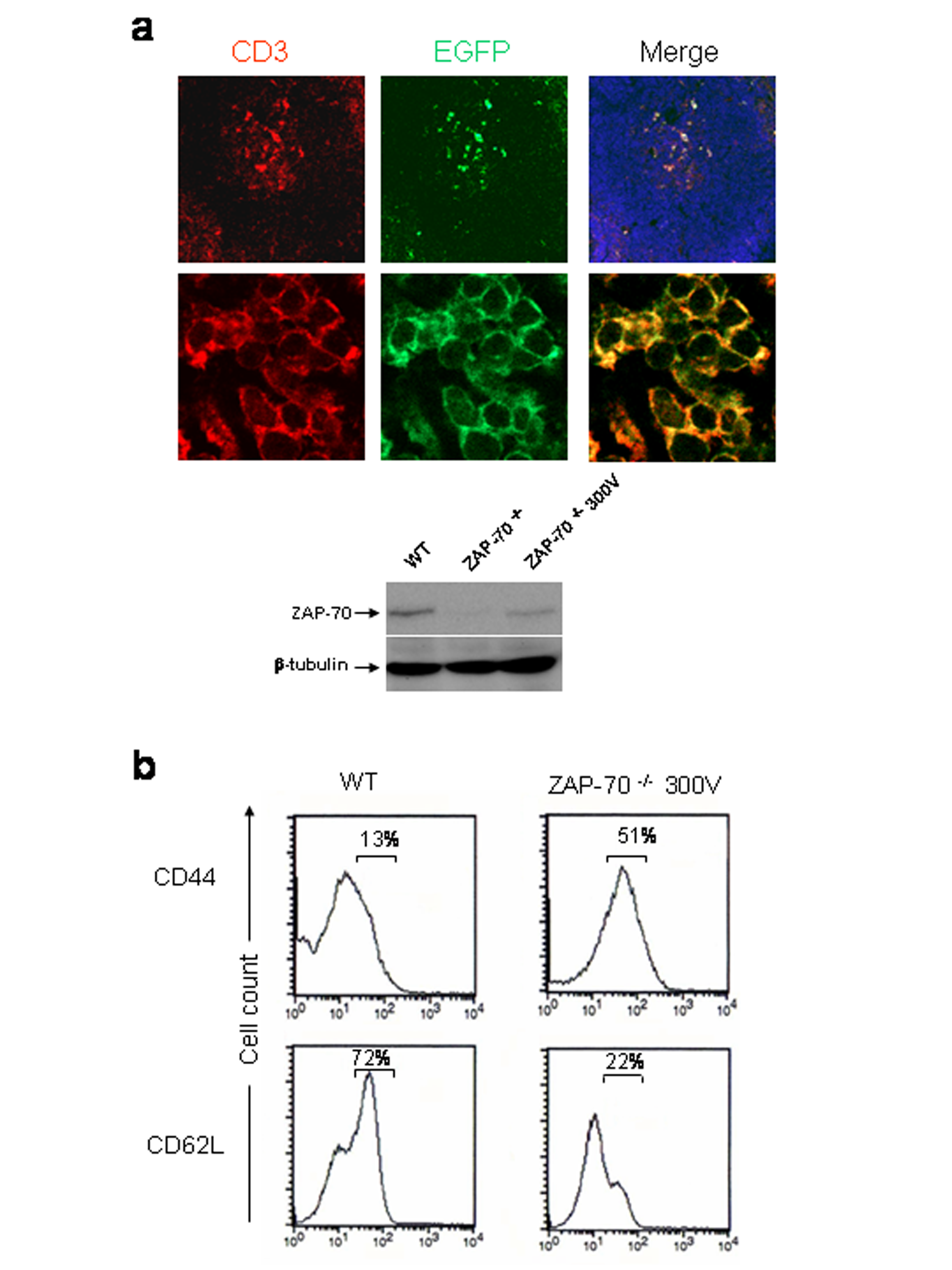

Supplement: Figure S3 — T lymphocytes exhibit an activated phenotype in reconstituted ZAP-70-/- mice. (A) The presence of EGFP T lymphocytes in reconstituted ZAP-70-/- mice was analyzed on splenic sections using a CD3 monoclonal antibody by confocal microscopy (upper panel). A nuclear counterstain (DAPI, blue) shows that transfected T lymphocytes are correctly localized in the white pulp (200X view). A higher magnification of transfected T lymphocytes was also presented (630X view). The lower panel shows a Western blot analysis of ZAP-70 protein levels in total thymocytes from spleens in WT, ZAP-70-/- deficient mice and in two weeks electroporated mice at 300 V (B) The phenotype of T lymphocytes was determined by using anti-CD62L and CD44 antibodies. The percentage of positively stained cells is indicated in each histogram. (2.63 MB TIF) [file pone.0002059.s003.tif]

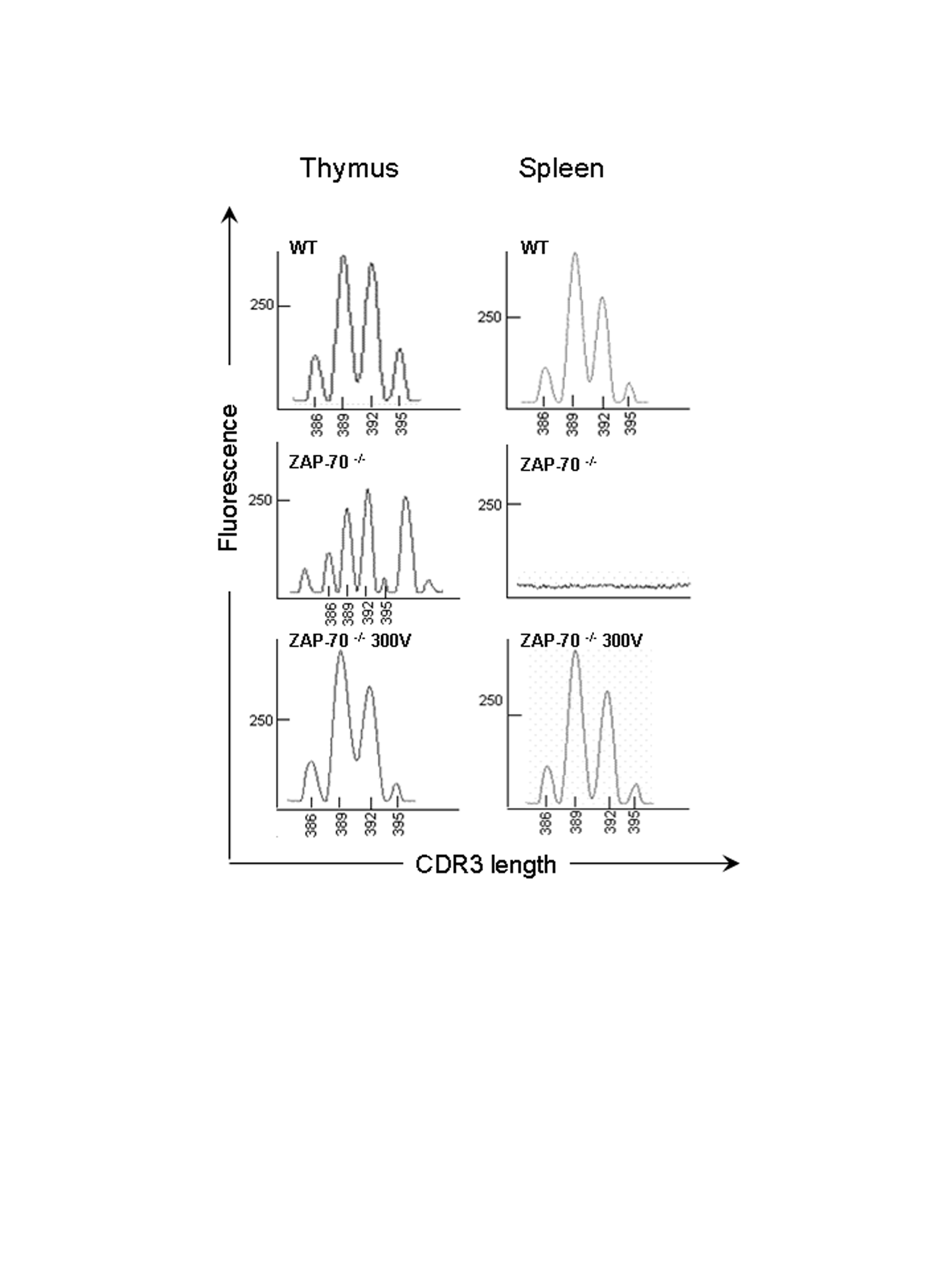

Supplement: Figure S4 — The sizes of the CDR3 were analysed by PCR followed by gel electrophoresis. The figure displays representative experiments of the CDR3 analysis of TCR transcripts for alpha chain bearing Valpha1. The relative intensity of the bands (y axis) was plotted as a function of the migration time in the electrophoresis (x axis) which is proportional to the size of CDR3. In each profile, the peaks corresponding to the reading frame are indicated. In WT, the spectral distribution of the sizes is representative of polyclonal composition of the TCR Valpha1 transcripts. In ZAP-70 deficient mice, no TCR transcripts are found in the spleen, whereas a few peaks of TCR Valpha1 transcripts are detected in the thymus, which are issued from non productive transcription known to take place in these mice. After electroporation of ZAP-70 plasmid (ZAP-70-/- 300 V), TCR transcripts bearing 1αV are detected in the thymus as well as in the spleen. The spectral distributions of the CDR3 sizes are, and on the one hand, similar in the thymus and the spleen of ZAP-70-/- 300 V electroporated mice, and on the other similar for WT and ZAP-70-/- 300 V electroporated mice. Thus, ZAP-70-/- 300 V electroporated mice are reconstituted with a polyclonal population of T cells which expressed a wide repertoire of TCR. (0.61 MB TIF) [file pone.0002059.s004.tif]

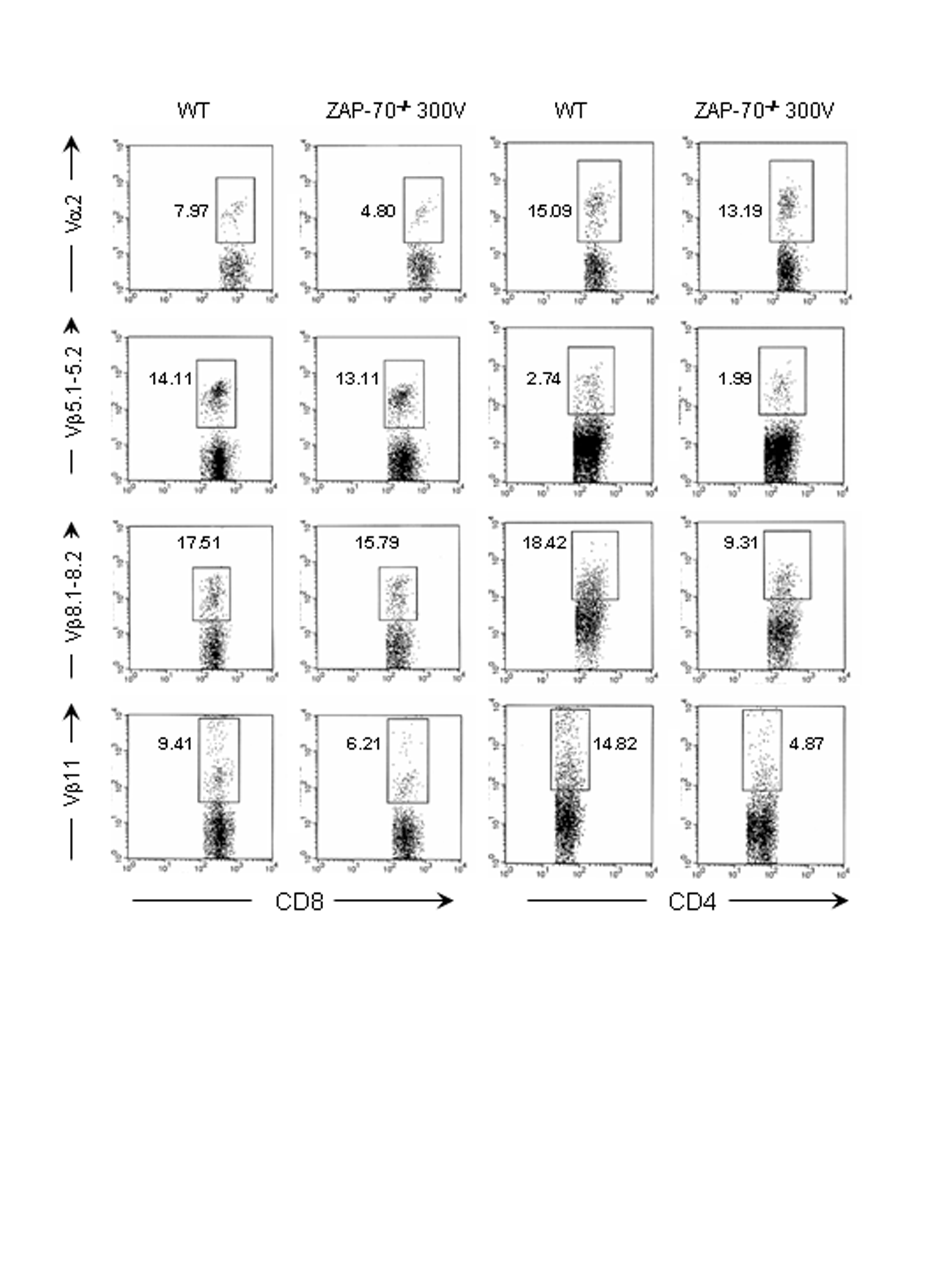

Supplement: Figure S5 — Representative dot plots of TCRVα2 and indicated TCRVβ usage in splenic CD8 and CD4 T cells populations. (1.57 MB TIF) [file pone.0002059.s005.tif]
